# Real-Time Pathogen Detection in the Era of Whole-Genome Sequencing and Big Data: Comparison of k-mer and Site-Based Methods for Inferring the Genetic Distances among Tens of Thousands of *Salmonella* Samples

**DOI:** 10.1371/journal.pone.0166162

**Published:** 2016-11-10

**Authors:** James B. Pettengill, Arthur W. Pightling, Joseph D. Baugher, Hugh Rand, Errol Strain

**Affiliations:** Biostatistics and Bioinformatics Staff, Center for Food Safety and Applied Nutrition, Food and Drug Administration, 5001 Campus Drive, College Park, MD 20740, United States of America; Indiana University Bloomington, UNITED STATES

## Abstract

The adoption of whole-genome sequencing within the public health realm for molecular characterization of bacterial pathogens has been followed by an increased emphasis on real-time detection of emerging outbreaks (e.g., food-borne Salmonellosis). In turn, large databases of whole-genome sequence data are being populated. These databases currently contain tens of thousands of samples and are expected to grow to hundreds of thousands within a few years. For these databases to be of optimal use one must be able to quickly interrogate them to accurately determine the genetic distances among a set of samples. Being able to do so is challenging due to both biological (evolutionary diverse samples) and computational (petabytes of sequence data) issues. We evaluated seven measures of genetic distance, which were estimated from either k-mer profiles (Jaccard, Euclidean, Manhattan, Mash Jaccard, and Mash distances) or nucleotide sites (NUCmer and an extended multi-locus sequence typing (MLST) scheme). When analyzing empirical data (whole-genome sequence data from 18,997 *Salmonella* isolates) there are features (e.g., genomic, assembly, and contamination) that cause distances inferred from k-mer profiles, which treat absent data as informative, to fail to accurately capture the distance between samples when compared to distances inferred from differences in nucleotide sites. Thus, site-based distances, like NUCmer and extended MLST, are superior in performance, but accessing the computing resources necessary to perform them may be challenging when analyzing large databases.

## Introduction

Whole-genome sequence (WGS) data provides a powerful means to discriminate among bacterial pathogens at a resolution not possible with other methods (e.g., pulse field gel electrophoresis) [1]. The utility and decreasing cost of obtaining WGS data has resulted in its quick adoption by public health agencies [2] and spurred initiatives for real-time pathogen detection in order to rapidly resolve outbreaks (e.g., [3]). One such initiative is the GenomeTrakr network, which consists of geographically distributed laboratories that are sequencing bacterial genomes (predominantly *Salmonella* and *Listeria*) and submitting the WGS data to the Sequence Read Archive (BioProject PRJNA183844), hosted by the National Center for Biotechnology Information. Since its inception in December 2012, the database has grown to include greater than 50K isolates.

The rapid growth of pathogen databases and the rate at which they are expected to grow, presents a unique set of challenges for the rapid detection of emerging outbreaks. In addition to infrastructure issues related to large amounts (i.e., petabytes) of sequence data, public health officials face the challenge of how best to analyze sequence data to obtain actionable information. Obtaining such information is nontrivial due to the need for determining the proper relationships of new samples to the existing set of samples. We envision a two-step process, which, ideally, would yield results in minutes or hours. The first step, which is the primary focus of the research presented here, is the approximation of the genetic distances among all samples (or between a focal sample and all others) in order to identify a subset of plausibly relevant samples. In step two, this subset of individuals would be investigated with a more robust method such as a reference-based approach to variant detection (e.g., [4]).

Here, we assess the efficacy of seven genetic distances (calculated from either k-mer profiles or nucleotide site differences) for accurately estimating the similarity among a diverse set of samples based on WGS data. We have focused on distances that could provide reliable identification of closely-related samples with an additional focus on computational efficiency, such that the method is scalable to datasets involving tens to hundreds of thousands of samples. Our list of methods evaluated excludes a number of open source site-based methods (e.g., kSNP [5] and Parsnp [6]) which do not scale even to the current number and evolutionary breadth of samples that exist in the rapidly growing GenomeTrakr databases.

## Materials and Methods

### Empirical dataset–*Salmonella* GenomeTrakr

All sequence data was obtained from the Sequence Read Archive (SRA) at the National Center for Biotechnology Information (NCBI) database. The reads were filtered for base quality (minimum of 80% at Q20), trimmed (first 15 positions), and size selected (> 50 bases) using the FASTX-Toolkit (http://hannonlab.cshl.edu/fastx_toolkit/). The remaining reads were filtered for contamination using Kraken [7]. This was accomplished by generating a custom Kraken database comprised of all *Salmonella* sequences present in NCBI’s RefSeq database and retaining only those reads which met a kraken-filter threshold of 0.25. For paired reads, both the forward and reverse reads were required to meet the threshold. We then performed *de novo* assemblies with SPAdes v3.6.1 [8], using default settings. We manually curated the metadata file associated with the database to remove samples with missing or uninformative serovar designations and to include those with SRA accessions. This reduced the number of samples from 27,631 to 18,997 (see S1 Table for metadata on samples analyzed).

### Measures of genetic distance

We inferred genetic distances using either differences in k-mer profiles or nucleotide sites (i.e., differences in aligned sequence data) (Table 1). Three of the distance measures estimated from k-mer profiles began with indexing the *de novo* assemblies into k-mers with jellyfish v2.0 [9],using k = 19 and the–C flag to obtain only canonical k-mers. Using these k-mer profiles, we calculated three pairwise measures of genetic distance: 1—Jaccard index, Manhattan, and Euclidean distances. We calculated all genetic distances based on k-mer profiles using custom python scripts. The two other distances estimated from k-mer profiles were computed with Mash [10], which took as input the *de novo* assemblies. These included an estimate of Jaccard distances (which we call Mash Jaccard) and Mash distances, both of which are based on comparisons of sketches (Table 1). We used a sketch size of 400 and k-mer length of 16. A sketch is a greatly reduced representation of the k-mer content within a genome that is obtained through a hashing algorithm; and see Ondov et al. 2015 for additional details. Exploratory analyses showed that increasing the sketch size did not alter the distances among samples. A k-mer length of 16, which is the default, was chosen as it represents the largest value that can be used without requiring a significant increase in disk space and memory.

**Table 1:** Summary of genetic distances used to infer the relationships among samples.

Class	Distance	Description
Site-based	NUCmer	Suffix array method to efficiently perform pairwise whole-genome alignment
	Extended MLST	Employs the Basic Local Alignment Search Tool to perform pairwise comparisons of predicted open reading frames
k-mer based	Jaccard Distance	1 –Jaccard index (i.e., the intersection divided by the union of all k-mers found between two samples)
	Manhattan Distance	Sum of the absolute differences between the abundance of each k-mer present between two samples
	Euclidean Distance	The square root of the sum of square of all pairwise differences in k-mer abundance
	Mash Distance	Employs the MinHash [23] technique to reduce genomes to sketches (i.e., a reduced representation of the information within the sequence data) and estimates a novel evolutionary distance metric among them
	Mash Jaccard Distance	The Jaccard Distance (as described above) but based on the sketch size (e.g., the number of hashes)

We also evaluated two genetic distance measures that were inferred from nucleotide sites (i.e., site-based), NUCmer (using the show-snps utility) within the MUMmer v3.0 package [11] and extended multi-locus sequence typing (MLST) (Table 1). With NUCmer, we calculated the pairwise SNP differences (i.e., the number of nucleotide sites differences) between samples by parsing the Variant Call Format (VCF) file and ignoring positions where gaps or missing data were inferred in one of the genomes.

For the extended MLST distance measure, we generated a set of target loci using methods described in Pightling, et al. 2015 [12]. Briefly, the core genome of *S*. *enterica* was calculated by downloading protein sequence predictions for 32 high-quality genomes from the NCBI. We then performed an all *versus* all pairwise alignment analysis using the Basic Local Alignment Search Tool (BLAST) [13]. Using BLAST to identify members of the core genome that reliably “hit” orthologs during both nucleotide and protein sequence bidirectional searches, we identified a total of 1,152 loci that are amenable to automated analyses. Allele profiles for previously unannotated genomes were generated by predicting open reading frames with Prokka [14] and identifying target loci with BLAST. Distances between profiles were determined by counting the total numbers of nucleotide mismatches across all alleles. Profiles were excluded on the basis of poor sequence assembly, as indicated by the presence of truncated or missing loci [15].

### Measure of performance

To evaluate the utility of the distance measures within the context of real-time pathogen detection, we used a classification approach using serovar as designated in the metadata file associated with the *Salmonella* samples in the GenomeTrakr database. We focused on the top 10 most abundant serovars (Table 2) among the 18,997 Salmonella samples. The classification was based on a one against-all scenario where a sample from each of the top 10 serovars was randomly selected and the genetic distance was calculated between that focal sample and all 18,996 other samples. Whether a sample to which the focal sample was being compared came from the same serovar or a different serovar determined whether the classification was IN or OUT, respectively. To account for variability in the similarity among members of the same serovar we performed 10 replicates where a random individual was chosen for each serovar for a total of 100 analyses under each distance type. We acknowledge that serovar designations do not always reflect evolutionary groups (e.g., Salmonella Newport is polyphyletic) [16, 17] and, thus, our use of serovar as the classifier will perform poorly in such instances (low sensitivity and specificity). However, this should affect all distance methods and, therefore, not introduce a misleading signal when evaluating the relative performance of the distance methods to one another (see Discussion for further details).

**Table 2:** Mean and variance of AUC values for each of the different distance methods for each serovar.

	Distance							
	k-mer based					Site-based		
Serovar (N)	Euclidean	Jaccard	Manhattan	Mash	Mash Jaccard	Extended MLST	NUCmer	Average
Agona (282)	0.767 (0.014)	0.849 (0.011)	0.822 (0.023)	0.935 (0.006)	0.944 (0.005)	0.985 (0)	0.959 (0.006)	0.894
Enteritidis (4455)	0.868 (0.002)	0.876 (0.003)	0.88 (0.003)	0.959 (0)	0.959 (0)	0.987 (0)	0.983 (0)	0.930
Heidelberg (580)	0.919 (0.001)	0.889 (0.002)	0.925 (0.001)	0.939 (0)	0.943 (0)	0.994 (0)	0.984 (0)	0.942
Infantis (341)	0.897 (0.002)	0.919 (0.001)	0.906 (0.001)	0.979 (0)	0.982 (0)	0.988 (0)	0.986 (0)	0.951
Kentucky (627)	0.709 (0.013)	0.749 (0.009)	0.756 (0.011)	0.875 (0.001)	0.872 (0.002)	0.968 (0)	0.936 (0)	0.838
Montevideo (287)	0.823 (0.002)	0.832 (0.003)	0.837 (0.004)	0.921 (0.005)	0.916 (0.007)	0.980 (0.001)	0.968 (0.001)	0.897
Newport (827)	0.680 (0.007)	0.677 (0.006)	0.680 (0.008)	0.819 (0.006)	0.813 (0.003)	0.976 (0)	0.948 (0)	0.799
Senftenberg (232)	0.793 (0.006)	0.821 (0.003)	0.814 (0.007)	0.925 (0.001)	0.933 (0.001)	0.974 (0)	0.958 (0)	0.888
Typhimurium (3475)	0.822 (0.003)	0.846 (0.003)	0.846 (0.003)	0.949 (0)	0.948 (0)	0.966 (0)	0.969 (0)	0.907
Weltevreden (268)	0.914 (0.002)	0.915 (0.001)	0.934 (0.001)	0.931 (0.001)	0.907 (0.001)	0.983 (0)	0.982 (0)	0.938
Average	0.819	0.837	0.84	0.923	0.922	0.980	0.967	

Based on the above classification scheme and genetic distances between focal samples and all others, we measured performance using receiver operator curves (ROCs) that plot the true-positive rate vs. false-positive rate. We also estimated the area under the curve (AUC) for each ROC, which ranges from 0 to 1 and represents the probability that a randomly selected sample from the same serovar is correctly ranked above another sample from a different serovar [18]. Analyses were performed using the python module scikit-learn. Results are based on a cross-validation procedure where the logistic model (describing the classification of samples under which pairwise distance was the predictor variable and IN/OUT was the binary response variable) was trained on 75% of the data and the remaining 25% of the data was used to evaluate model performance.

Given the fact that the extended MLST method excluded some comparisons, we also evaluated the performance of the other measures on only those samples found in the extended MLST analyses. The results changed in the expected direction as there was an improvement in performance on the reduced dataset but only by an average of 1.6%. As a result, we present figures based on all samples that could be analyzed under each distance method.

### Estimates of computational demands

As a means of further evaluating the utility of the distance measures, we also captured the computational time necessary to estimate them. The fact that databases will grow to hundreds of thousands of samples makes runtime a very important factor to consider when choosing a method. The Jaccard, Manhattan, and Euclidean distance measures were coded in custom python scripts; extended MLST was coded in custom Perl scripts; the remaining distance measures were calculated using software developed outside our group. Estimates of runtime were acquired using the python timeit function. We acknowledge that these estimates of runtime are approximate as they do not account for other processes running and, with respect to the distance measures we coded, there are likely faster implementations of them that could be written.

## Results and Discussion

### Empirical data

**Fig 1:**
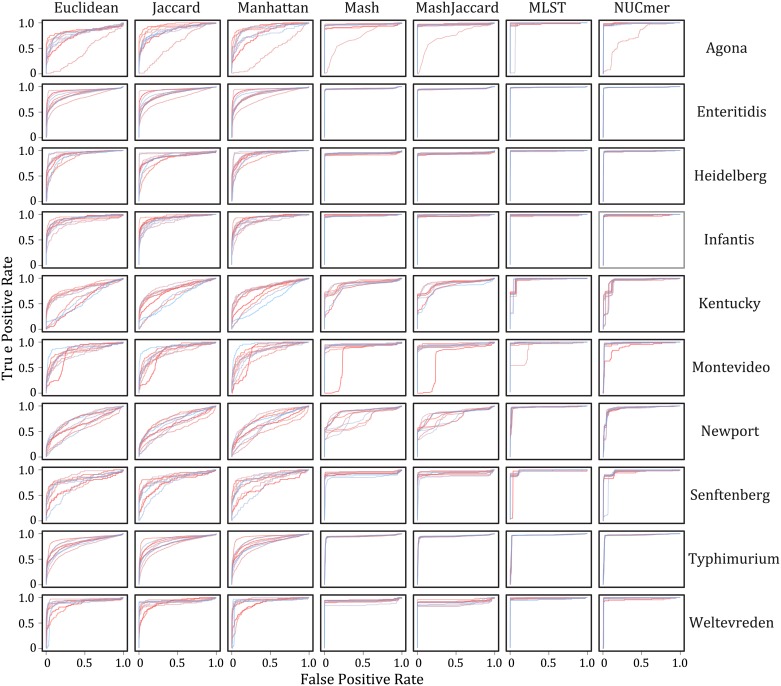
Receiver operator curves for each distance (columns) by serovar (rows) combination. Lines within each panel represent the 100 replicate analyses performed.

Based on the analysis of the WGS data for *Salmonella*, we found that certain distance measures performed much better than others and this pattern was consistent across serovars (Fig 1). The mean and variance of the AUC values are presented in Table 2. The best performing distance measure was calculated from the site-based extended MLST method, which had a mean AUC value across all replicates and serovars of 0.980. The next best method was NUCmer, which is also site-based and had an average AUC value of 0.967. Mash and Mash Jaccard had an average AUC of 0.923 and 0.922, respectively, and were the next best performing. The other three k-mer based methods (Euclidean, Manhattan, and Jaccard) all had mean AUC values < 0.85. Interestingly, the additional information content (e.g., abundance not just presence/absence of k-mers) entailed in calculating the Manhattan and Euclidean distance metrics did not result in a difference in performance compared with the Jaccard distance.

The ability to correctly differentiate IN samples from OUT samples varied across the different serovars. For example, serovars Heidelberg, Enteritidis, Infantis, Typhimurium, and Weltevreden had an average AUC value > 0.9 across the different distance methods. This in contrast to the serovar Newport, which had an average AUC value of 0.761. This is not surprising given that Newport is polyphyletic with two distinctly divergent clades. Within certain serovars (e.g., Agona and Montevideo; Fig 1) there also appear to be ‘outlier’ samples that result in poor performance of the different distance methods. Potential explanations for this are that the serovar of the randomly chosen focal sample was incorrectly assigned, the sample is heavily contaminated (albeit with congeneric material as we filtered sequences not belonging to the *Salmonella* genus with Kraken), or that there are certain samples with the correct serovar designation but that designation does not accurately reflect phylogeny [16, 17]. However, all distances should be affected equally by these artifacts; there will be both a larger number of k-mers or nucleotide sites that differ between genomes that are incorrectly classified as the same serovar than what is expected based on other pairs that are correctly classified.

### k-mer methods: treating missing data as informative data

The primary explanation for the difference in performance between distances calculated based on nucleotide site differences and k-mer-profiles is likely how the latter treats missing data (or absent k-mers) as informative; the distances based on sites, as we implemented them, ignore such artifacts. A longer k-mer length would further exacerbate this problem whereas decreasing the k-mer length would ameliorate it but at the expense of decreasing the ability to detect differences that exist among samples. To explain this, consider two identical genomes that only differ in that one has a number of bases deleted relative to the other. Their dissimilarity will be nonzero when a k-mer-based method is applied but zero when a site-based method is applied because ‘gaps’ are treated as missing (i.e., uninformative) data. As a result, poorly filtered, contaminated, erroneously assembled contigs, and/or mobile elements that are not part of the bacterial genome will all carry discriminatory information under k-mer-based methods. This phenomenon is well illustrated in the difference between the Jaccard distance we estimated and the Mash Jaccard distance. Within our estimate we did not filter k-mers based on their presence across samples within the dataset, thus, there are likely to be many singletons (k-mers found in only a single individual) that will drive up distance measures. In contrast, Mash Jaccard distance (and Mash distance) probabilistically filters k-mers found only once in a dataset using a Bloom filter.

The issue of treating missing data, due to erroneous assembly artifacts or contamination, as informative raises the possibility that the distance measures may perform quite similarly when working with high quality, closed genomes or heavily curated draft assemblies. Preliminary results of simulated data suggest this is likely to be the case. Those simulations under the coalescent using the ms [19] program, seq-gen to simulate sequences of 3 Mbp under the HKY [20] model of sequence evolution, and ART [21] as a means to introduce more realism into the analyses, showed that methods performed nearly identically with an AUC value of 1 or at least 0.99. However, even more robust draft or closed genomes will likely differ in gene content and mobile elements and, thus, k-mer and site-based methods will differ. Different organisms are also known to vary greatly in the number of genes shared or differing among individuals and those taxa that have open or unbounded pan genomes (e.g., Streptococcus pneumoniae and Escherichia coli; [22]) This raises a larger issue beyond the scope of the work presented here, which is whether missing data that can be confidently assigned to represent differences in evolutionary history should be coded as informative (e.g., a fifth state).

### Computational issues

**Fig 2:**
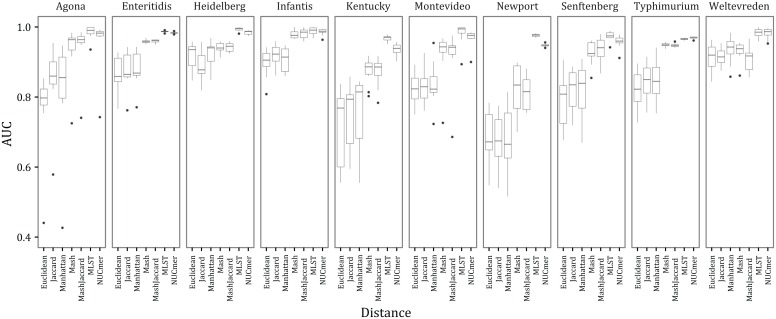
Boxplots illustrating the variation in area under the curve (AUC) values across the distance by serovar combinations. Note the minimum on the y-axis if 0.4.

The non-Mash distance measures were calculated on a heterogeneous high performance computing (HPC) cluster, where often hundreds of pairwise distance measures were being calculated simultaneously. For the Jaccard, Manhattan, and Euclidean distances estimated from k-mer profiles, there is little need to consider the computational resources necessary to efficiently calculate them (which are the greatest among the methods investigated; Table 3) as those distance measures perform poorly (Figs 1 & 2). Furthermore, they require the indexing of the genome into k-mers, which takes a nontrivial amount of time for tens of thousands of samples (Table 3).

**Table 3:** Median runtime estimates in seconds.

Class	Distance	Median
k-mer based	Euclidean*	53.123
	Jaccard*	2.781
	JellyFish^¶^	118.472
	Manhattan*	53.454
	Mash and Mash Jaccard^§^	1.226
Site-based	Extended MLST*	46.950
	NUCmer*	9.425

* For a single pairwise comparison

^¶^ To count and dump a text file of k-mers per sample

^§^ Includes the sketching of the focal sample and estimating the distance between it and all other samples

In contrast, Mash, extended MLST, and NUCmer do not require a prior indexing of the genome into k-mers. NUCmer, which was the second best performing measure, is a pairwise comparison and, thus, takes substantially more time than the Mash analyses. The latter is extremely fast where there is no need for substantial computing resources as the sketching and distance calculation of a focal sample against all other samples takes a second or two (Table 3) (that estimate does not include the sketching of the 18,779 samples, which takes a few hours) on a single workstation with 124 GB of RAM and 1.2 GHz processor. Our results suggest that with NUCmer one could obtain the estimates of distance between a focal sample and ~20,000 other *de novo* assemblies in under an hour if running 100 pairwise comparisons simultaneously (albeit on an HPC or powerful desktop machine).

Similar to the Jaccard, Manhattan, and Euclidean distance methods, extended MLST requires significant preparation of sequence data by performing open reading frame annotation prior to analyses. However, as extended MLST was shown here to provide the best estimates of distance between focal samples, the extra computational investment may be worthwhile as that method also provides additional information amenable to typing and functional genomic differentiation.

### Pathogen detection in practice and the consequences of different distances

In practice, real-time pathogen surveillance entails both the rapid population of databases and subsequent rapid bioinformatics analyses to detect epidemiologically relevant events. One scenario is that a practitioner will want to know all samples in a given database that are sufficiently close enough to a focal individual to warrant further investigation (e.g., with a more robust reference-based method for identifying genomic differences like the CFSAN SNP Pipeline [4]. Mash certainly seems to be a feasible method as it is extremely fast, requires little computational overhead, and easily accommodates new samples. However, the Mash method suffers from relying on k-mers where missing data is treated as informative and, thus, will result in biased distance estimates if *de novo* assemblies are incomplete, contain mobile elements, or are contaminated; these artifacts will result in an increase in false negatives (samples will appear more distant to one another than they really are). The MinHash method also decreases the specificity [10], which in practice, will hamper analyses with potentially irrelevant samples,which may be less of a concern than false negatives. NUCmer avoids many of the issues with k-mers, but being site-based where missing data is ignored, can lead to high false positive rates if additional information is not incorporated (e.g., proportion of the genome aligned). Also, unlike Mash, NUCmer does not scale well to obtaining an all-against-all measure of genetic distance, as the analyses here were based on the scenario in which a new sample is investigated by comparing to all others.

Focusing on the AUC values, the differences in performance among the distance measures may have important consequences in an outbreak investigation. For example, under the poor performing Manhattan distance there is only an approximately 84% chance that a randomly selected sample from the same serovar will be correctly ranked higher than a sample from a different serovar and this probability is only 92% for the best performing k-mer based method (MASH; Table 2). In practice, these results suggest that identification of samples close to a focal clinical sample (for example) will be incorrect perhaps 10% or more of the time. In contrast, extended MLST and NUCmer had 98% and 97% probabilities, respectively, of correctly assigning a randomly chosen sample from the same serovar as one randomly chosen from a different serovar.

## Conclusions

To achieve effective, real-time pathogen surveillance, it is paramount to be able to accurately determine the relationships among a diverse and large whole-genome sequence database of bacterial pathogens and to identify those samples that are genetically similar to newly sequenced samples. We have evaluated a suite of different genetic distances and the results indicate that there is a real benefit to the use of those inferred from nucleotide site differences (e.g., NUCmer or extended MLST) over those inferred from k-mer profiles. Therefore, though distances inferred from k-mer profiles do provide results faster, in order to reduce the potential for false negatives caution should be taken when relying on such distances.

## Supporting Information

S1 Table Supplemental Table.tsv. Metadata, including SRA accessions for the sample analyzed.
(TSV)
